# Sex-Specific Prevalence of Diabetes and Cardiovascular Risk Factors in the Middle-Aged Population of China: A Subgroup Analysis of the 2007–2008 China National Diabetes and Metabolic Disorders Study

**DOI:** 10.1371/journal.pone.0139039

**Published:** 2015-09-25

**Authors:** Shi Bu, Danjie Ruan, Zhaojun Yang, Xiaoyan Xing, Wenhui Zhao, Na Wang, Lingding Xie, Wenying Yang

**Affiliations:** 1 Department of Endocrinology, China–Japan Friendship Hospital, Beijing 100029, PR China; 2 Department of Endocrinology, the 1^st^ Hospital of Huairou District, Beijing 101400, PR China; University of Bologna, ITALY

## Abstract

The sex difference in the prevalence rates of diabetes and cardiovascular diseases (CVDs) among the middle-aged population in China remain largely unknown. Therefore, we analyzed differences in the prevalence of diabetes, self-reported CVDs, and some CVD risk factors among men and women in the middle-aged population (30–49 years) and in individuals aged 50 years and older using data from the China National Diabetes and Metabolic Disorders Study of 2007–2008. Middle-aged men appeared to have significantly a higher prevalence of diabetes and self-reported CVDs than middle-aged women (8.07% *vs* 5.06% for diabetes, P < 0.001; 0.64% *vs* 0.22% for CVDs, P < 0.001). Men also showed higher rates of central obesity, hypertension, and dyslipidemia than women (all P < 0.01). Compared with women, men were more likely to drink alcohol and smoke cigarettes but less likely to be under diet control. The sex-specific differences in prediabetes, CVD, and CVD risk factors between men and women were diminished or even reversed in the population aged 50 years and older. No sex-specific differences were found in the prevalences of a family history of diabetes, coronary heart disease, and hypertension (P > 0.05) in middle-aged population. Specific strategies to reduce modifiable risk factors for the prevention and control of diabetes and CVD may be warranted in this population.

## Introduction

Sex-specific differences in the prevalence of diabetes are unclear except for a few specific types of diabetes. The China National Diabetes and Metabolic Disorders Study [1], a recent epidemiological study of the Chinese population using a nationally representative sample, has reported a higher prevalence of diabetes in men (5.2%) than in women (3.0%) among participants aged 30–39 years and a consistently higher prevalence in men (11.1%) aged 40–49 years compared to their female counterparts (7.3%). A more recent study in the Chinese population also has indicated that in the population younger than 50 years, men are more likely to be diagnosed with diabetes than women, and thus, being male is a risk factor for diabetes in this population [2]. Previous surveys conducted in developed countries such as Canada and Australia have reported the same results. In contrast, being male was not a risk factor for diabetes among the Chinese population in a national population-based survey in 1994[3]. Interestingly, only 4 years later in 1998, another national survey reported higher prevalence rates for both diabetes and impaired glucose tolerance in women than in men[4]. Thus, there is an urgent need to clarify the reason for this change in the sex-specific prevalence of diabetes.

Cardiovascular diseases (CVDs) represent a major cause of death in patients with type 2 diabetes. More than 80% of CVD- and diabetes-related deaths occur in low- and middle-income countries. China is still among the low- and middle-income countries and is home to the largest absolute number of diabetes patients. The prevention and control of diabetes and related CVDs have posed a serious challenge to the country. Data on the prevalence of diabetes in men and women have been made available through previous epidemiological studies. However, no study has elucidated the factors underlying the discrepancy in the prevalence between men and women in the middle-aged population. In the present study, using data from the 2007–2008 China National Diabetes and Metabolic Disorders Study, we aimed to analyze the differences in prevalence rates of diabetes, CVDs, and CVD risk factors between middle-aged men and women, in order to support the development of tailored measures for diabetes and CVD prevention and control in this working-age population.

## Methods

### Study population

A population-based cross-sectional survey entitled “the China National Diabetes and Metabolic Disorders Study” was carried out from June 2007 to May 2008. The details of population sampling have been described previously[1]. Through a multistage, stratified random sampling process, individuals from a total of 152 urban street districts and 112 rural villages were selected. Among these districts and villages, 54,240 people aged 20 years or older had lived at their current residence for 5 years or longer and were thus eligible for inclusion in the study. Among them, 39,071 individuals aged 30 years and older (15,299 men and 23,772 women) had provided the complete data required for the current analysis.

### Ethics statement

The Clinical Research Ethics Committee of China-Japan Friendship Hospital reviewed and approved the study program (No. 2007–026). Each participant signed an informed consent form before data collection.

### Screening strategies for diabetes and CVD risk factors

Each participant was interviewed by trained and qualified physicians. Data regarding demographic characteristics, lifestyle risk factors, and personal and family medical histories were collected using a standard questionnaire[1]. The interview included questions related to the diagnosis and treatment of diabetes, hypertension, dyslipidemia, and cardiovascular events. Previously diagnosed non-fatal CVDs were determined by self-reporting. If the participant self-reported a history of hospitalization for myocardial infarction or coronary balloon angioplasty or a history of coronary stent implantation or coronary bypass surgery, he/she was defined as having coronary heart disease (CHD). Stroke was defined if a continued language or limb movement disorder persisted for more than 24 hours, or if a participant had received a diagnosis of ischemic or hemorrhagic stroke supported by computed tomography (CT) or magnetic resonance imaging (MRI). Both CHD and/or stroke were recorded as CVDs. Participants who had smoked at least 100 cigarettes in one's lifetime were defined as smokers. Participants who had drunk at least 30 g of alcohol per week for 1 year or more were defined as alcohol drinkers. Participants who participated in moderate or vigorous activity for at least 30 minutes per day at least 3 days per week were defined as having regular leisure-time physical activity. Socioeconomic status, educational level, occupation, and income also were included in the questionnaire.

Blood pressure, body weight, height, and waist circumference were measured using standard methods described previously[1]. Waist circumference was measured at the minimal horizontal girth between the rib cage and iliac crest, and the hip circumference was measured at the maximal horizontal girth between the waist and thigh. Central obesity was defined as a waist circumference ≥90 cm for men and ≥85 cm for women. Right upper arm blood pressure was measured twice with an interval of 30 s in a sitting position using a mercury sphygmomanometer, and the average of the two readings was used in the current data analysis. Hypertension was defined as an average blood pressure ≥140/90 mmHg or a previous history of hypertension. Standard methods for the oral glucose tolerance test (OGTT) and measurement of lipids and glucose also have been described in detail previously [1].

### Definitions of diabetes and prediabetes

Previously diagnosed diabetes was defined by self-reporting of a prior history of diabetes, which was confirmed by a doctor according to a previous fasting plasma glucose level ≥7.0 mmol/L and⁄or information on glucose-lowering treatment regardless of the fasting plasma glucose level. Individuals without a history of diabetes were classified as having diabetes if their fasting plasma glucose was ≥7.0 mmol/L and⁄or the 2-h plasma glucose was ≥11.1mmol/L. Prediabetes was defined by a fasting plasma glucose of ≥6.1 mmol⁄L and <7.0 mmol⁄L and/or a 2-h glucose ≥7.8 and <11.1 mmol/L[5].

### Statistical analysis

Statistical analyses were conducted using the SUDAAN software (version 10; Research Triangle Institute, Research Triangle Park, NC, USA). All calculations were weighted to represent the total Chinese adult population aged 20 years or older. Standard prevalence rates were calculated based on the overall population according to age, and the age-standardized prevalence was determined using the population distribution of China reported by the National Bureau of Statistics of China in 2006.

The data are presented as means with 95% confidence intervals (CIs). The differences between group means and between-group frequencies were tested using a ‘PAIRWISE’ procedure in the SUDAAN software. The test for trends employed a polynominal contrast procedure. Variables without normal distribution were transformed logarithmically before analysis. A multiple logistic regression analysis was employed to examine the association between diabetes prevalence and possible risk factors, and adjusted odd ratios were obtained. All P-values were two-tailed, and a P-value of <0.05 was considered statistically significant.

## Results

### General participant characteristics

Table 1 shows comparisons of the prevalence rates of diabetes, prediabetes, self-reported CVDs, and CVD risk factors between men and women aged ≥30 years. The prevalence of diabetes in middle-aged (aged 30–49 years) men was significantly higher than that in women of the same age group (8.1% v.s. 5.1%, P < 0.001). The prevalence of self-reported CVD for men was 3-fold that for women in this population (0.6% v.s. 0.2%, P = 0.001). In addition, among participants aged 30–49 years, the prevalence of prediabetes was higher in men than in women, as were the prevalence rates of most of the CVD risk factors such as body mass index (BMI), waist circumference, waist-to-hip ratio, central obesity, hypertension, high blood pressure, and elevated plasma levels of triglycerides, total cholesterol, and LDL-cholesterol. The differences in the prevalence rates of impaired glucose tolerance, CVD, and CVD risk factors between men and women were diminished or even reversed in the population aged 50 years and older.

**Table 1:** Clinical characteristics of the study sample by age group and sex.

	Aged 30–49 years		Aged ≥50 years	
	Men	Women	Men	Women
Number	8352	13419	6947	10353
Diabetes (yes, %)	8.1	5.1	17.6	17.1
	7.27,8.94^‡^	4.53,5.06	16.06,19.14	15.61,18.71
Prediabetes ^a^ (yes, %)	14.9	12.5	21.6	22.6
	13.68,16.19^†^	11.65,13.30	19.89,23.30	20.56,24.69
Self-reportedCVD prevalence ^b^ (yes, %)	0.64	0.22	4.04	2.76
	0.45,0.91^†^	0.14,0.37	3.35,4.87^†^	2.09,3.63
Fasting plasma glucose (mmol/L)	5.24	5.08	5.59	5.63
	5.19,5.28^‡^	5.05,5.11	5.52,5.66	5.49,5.76
2-h plasma glucose (mmol/L)	6.53	6.42	7.89	8.19
	6.43,6.63	6.35,6.49	7.73,8.06^†^	7.96,8.41
Body mass index (kg/m^2^)	24.3	23.5	24.1	24.3
	24.2,24.5^‡^	23.4,23.6	24.0,24.3	24.1,24.4
Waist circumference (cm)	84.1	76.8	84.6	82.5
	83.8,84.5^‡^	76.6,77.1	84.2,85.0^‡^	82.0,83.0
Central obesity ^c^ (yes, %)	29.6	18.7	33.4	41.8
	28.1,31.1^‡^	17.7,19.7	31.6,35.3^‡^	39.6,44.0
Waist-hip ratio	0.88	0.82	0.89	0.87
	0.88,0.88^‡^	0.82,0.83	0.88,0.89^‡^	0.86,0.87
Total cholesterol (mmol/L)	4.73	4.55	4.83	5.18
	4.69,4.76^‡^	4.52,4.57	4.80,4.87^‡^	5.14,5.21
Triglycerides (mmol/L)	1.87	1.31	1.64	1.71
	1.82,1.92^‡^	1.28,1.33	1.59,1.68^‡^	1.67,1.745
HDL-c (mmol/L)	1.24	1.34	1.27	1.36
	1.22,1.25^‡^	1.33,1.35	1.26,1.29^†^	1.35,1.38
LDL-c (mmol/L)	2.66	2.53	2.79	2.98
	2.63,2.69^‡^	2.50,2.55	2.75,2.82^‡^	2.93,3.02
Hypertension (yes, %)	24.4	14.8	46.8	47.8
	23.0,25.8^‡^	13.9,15.7	44.8,48.8	45.6,50.1
SBP (mmHg)	121	115	131	132
	120,121 ^‡^	115,116	130,132	131,133
DBP (mmHg)	79	75	81.2	79.3
	79,80^‡^	75,76	80.7,81.7^‡^	78.8,79.8

variables are presented as the mean with 95% CI.

^†^ P < 0.05

^‡^ P < 0.001

SBP, systolic blood pressure. DBP, diastolic blood pressure.

^a^ Prediabetes was defined according to the 2010 American Diabetes Association criteria.

^b^ Cardiovascular diseases (CVDs) were defined including myocardial infarction, balloon angioplasty and cardiac stenting, and coronary artery bypass surgery.

^c^ Definition of central obesity: waist circumference ≥90 cm for men and ≥85 cm for women.

### Lifestyle and social-economic status

The differences in lifestyles and socioeconomic status between middle-aged men and women are presented in Table 2. Men were more likely to smoke and be alcohol drinkers than women. On the other hand, more women than men were consciously under diet control. No difference in the proportions of individuals who participated in physical exercise was found between men and women. About 80% of both men and women aged 30–49 years had attained secondary or higher education. More women than men had attained only primary education among participants aged 50 years and above. Finally, a higher proportion of men than women earned an annual personal income of ≥30,000 RMB.

**Table 2:** Lifestyle and socioeconomic status of the study sample by age and sex.

	Aged 30–49 years		Aged ≥50 years	
	Men	Women	Men	Women
Number	8352	13419	6947	10353
Alcohol drinking (yes, %)	49.77	4.47	36.13	3.64
	48.00,51.54 ^‡^	3.94,5.08	34.22,38.09^‡^	2.99,4.41
Smoking (including current and ex-smokers) (yes, %)	54.79	2.14	47.19	4.74
	53.03,56.55^‡^	1.80,2.54	45.17,49.23^‡^	3.98,5.65
Physical activity (yes, %)	28.85	27.71	38.84	38.38
	27.28,30.48	26.52,28.93	36.92,40.80	36.24,40.56
Diet control (yes, %)	14.45	17.33	23.03	25.85
	13.33,15.65 ^‡^	16.38,18.33	21.39,24.76^†^	23.97,27.83
Education levels (yes, %)				
Primary	14.30	22.48	41.73	61.91
	12.97,15.75^‡^	21.24,23.77	39.70,43.80^‡^	59.96,63.83
Secondary	59.52	59.97	46.17	33.42
	57.76,61.26	58.58,61.35	44.18,48.18^‡^	31.64,35.25
Tertiary or above	26.17	17.55	12.09	4.67
	24.70,27.70^‡^	16.58,18.56	10.93,13.36^‡^	4.00,5.45
Personal income per year (RMB ^a^, %)				
RMB 30,000–100,000	19.21	13.95	13.66	11.72
	17.88,20.61^‡^	13.04,14.92	12.33,15.12^†^	10.67,12.87
< RMB 30,000	78.89	84.90	85.30	87.06
	77.43,80.27^‡^	83.90,85.84	83.80,86.68	85.44,88.52

variables are presented as the mean with 95% CI.

^†^ P < 0.05

^‡^ P < 0.001

^a^ 1 USD = 6.2 RMB

### Multivariable risk assessment

In the multivariable regression model, male sex, older age, family history of diabetes, higher BMI, elevated systolic blood pressure, elevated serum triglyceride level, and non-tertiary educational level were significantly associated with an increased risk of diabetes in the middle-aged population (Table 3).

**Table 3:** Odds ratios (95% confidence intervals) for diabetes corresponding to measured variables.

Variable	Odds ratio (95% CI)	P value
Sex (men *vs* women)	1.45 (1.19–1.76)	<0.001
Family history of diabetes (yes *vs* no)	2.92 (2.40–3.56)	<0.001
Education level		
Tertiary or above	1 (Reference)	
High school	1.42 (1.13–1.80)	<0.001
Elementary school	1.52 (1.08–2.14)	<0.001
TG per increase of 0.56mM (50mg/dl)	1.12 (1.09–1.16)	<0.001
Body mass index (kg/m^2^)	1.12 (1.09–1.15)	<0.001
Systolic blood pressure, per increase of 10 mmHg	1.23 (1.16–1.29)	<0.001
Age, per 10-yr increment	2.09 (1.73–2.52)	<0.001

Logistic regression model adjusted for cigarette smoking and alcohol consumption, level of leisure-time physical activity, serum cholesterol, resident area, and personal income in step 1. Because the aforementioned variables were not significantly associated with diabetes, we excluded them in the final model. In step 2, all variables fitted into the final model are presented in Table 3.

### Prevalence rates of diabetes, self-reported CVDs, and CVD risk factors in the middle-aged population

We further stratified the middle-aged group into two age-based subgroups of 30–39 years (3971 men and 6286 women) and 40–49 years (4349 men and 7076 women) and compared the prevalence rates of diabetes, CVDs, and CVD risk factors between these groups (S1 Fig). The prevalence rates of diabetes, CVDs, hypertension, overweight or obesity, central obesity, and hypertriglyceridemia were higher among men than women in both subgroups (30–39 vs. 40–49 years, all P < 0.05). However, no differences in family history of diabetes, acute myocardial infarction, stroke, and hypertension were detected between men and women in these subgroups (all P > 0.05).

## Discussion

In this nationwide population-based cross-sectional study of the Chinese population, we found that the prevalence of diabetes, self-reported CVDs, and CVD risk factors were remarkably higher in middle-aged men than women. However, among those aged 50 years and older, this sex-specific difference weakened to a non-significant level or even reversed. Being male and having a family history of diabetes, high BMI, elevated serum triglycerides, and higher systolic blood pressure were associated with diabetes in middle-aged individuals (30–49 years old).

Previous studies have shown these sex-specific differences before. In the AusDiab study, which was conducted in 1999–2000, men were found to have a higher prevalence of diabetes (8.0%) than women (6.8%), and the sex-specific difference was most notable in those aged 45–64 years[6]. Men at an average age of 50 years were more likely to report known diabetes and a higher fasting glucose level than women in a population-based survey in Sweden [7]. In contrast, in some particular races, women are more likely to develop diabetes than men. A systematic review included 11 studies of the prevalence of diabetes in Australian Aborigines [8], and nine of these studies reported a higher prevalence of diabetes in women (9.3%–33.0%) than men (7.5%–18.9%). Three studies demonstrated that women have a higher prevalence of impaired glucose tolerance (5.2%–15.7%) than men (4.2%–13.9%) [8]. A plausible explanation could be that indigenous women were more likely to be obese than indigenous men in most of these studies. Data from the Canadian National Population Health Survey conducted in 1996–1997 also confirmed this sex difference. A Canadian study found that a low socio-economic status was significantly associated with self-reported diabetes in women but not in men for those aged 40 years and older[9].

A national epidemiological survey on diabetes was conducted in China in 1994. It covered a random sample of 224,251 participants aged 25–64 years from 19 provinces and regions in China[4]. Although different methodology and a different definition of diabetes were adopted in this survey in comparison to other studies conducted later, higher trends for the prevalence of diabetes were found in women than men aged 25–64 years (2.40% [95% CI 2.31–2.49] in women vs. 2.21% [95% CI 2.12%–2.30%] in men) [4]. The present study conducted in 2007–2008 showed a higher prevalence of diabetes in middle-aged Chinese men compared to women. The sex difference in the prevalence of diabetes in the same ethnic population over different eras cannot be fully explained by genetics or sex hormones. It may simply be a temporary phenomenon related to the socio-economic development during a particular historical period of China.

Our study also found that more middle-aged men suffered from central obesity, hypertriglyceridemia, high blood pressure, or hypercholesterolemia than women of the same age. This finding is consistent with the results of previous population-based studies in Taiwan Chinese and Swedish populations [10]. A plausible interpretation is that the rapid development of China's economy in the past two decades may have led to various changes in lifestyles for men and women. The modernization of production technologies has contributed to a dramatic reduction in the intensity of labor among industrial workers and agricultural laborers, the majority of which are men in China. Social and economic development has changed the lifestyle of most middle-aged men, particularly those living in urban areas. The westernization and/or urbanization of lifestyle, such as a transition to a more sedentary lifestyle and increased access to food at an increased number of social events, are associated with a high risk for obesity, diabetes, and other metabolic disorders. Moreover, middle-aged men have more chances than women to drive a car and attend banquets, a most common form of social activity in China, but are less likely to perform physical exercise.

It is notable that men aged 30–49 years appeared to have higher prevalence of diabetes and self-reported CVDs than women in this age range. Further analysis did not show any difference between men and women regarding the prevalence of a family history of diabetes, CVD, or hypertension. This suggests that the sex-specific differences in the prevalence rates of diabetes and self-reported CVDs may not be related to genetic factors. On the other hand, modifiable CVD risk factors such as smoking, lack of physical activity,[11] obesity, dyslipidemia, and hypertension arise more frequently in men than in women. This phenomenon provides healthcare professionals with a potential opportunity to control the epidemic of diabetes and CVDs by reducing modifiable risk factors in middle-aged Chinese men.

Given the sex-specific differences in social psychological characteristics between men and women, diabetes prevention strategies tailored specifically for men are needed. Davies and colleagues reported that male college students are more inclined to maintain an independent social image and pay less attention to the impact of their current behavior on their future health status[12]. Men are more likely to neglect potential health problems such as obesity and to underestimate the necessity of seeking medical advice and services. Consequently, these social psychological characteristics increase their risk for diabetes. Additional evidence of behavior-related disparities between men and women has been provided by disease prevention programs in middle-aged and elderly in Taiwan [13]. A series of disease prevention activities (including those for diabetes) and health monitoring preferential policies, such as one free health examine every 3 years for those aged 40–65 years, have resulted in more women than men accessing these health care resources. An interesting finding is that the prevalence of diabetes increased from 9.34% in the years 1993–1996 to 18.44% in 2005–2008 in middle-aged men, while the corresponding figures for middle-aged women dropped from 13.98% to 11.1%, according to a comparison of the findings of two diabetes epidemiological surveys conducted before and after this health promotion campaign in Taiwan[10].

The present study provides clues regarding the sex difference in the prevalence rates of diabetes and cardiovascular risk factors among the middle-aged Chinese population. Given the rapid modernization of Asia and many cultural similarities between China and other Asian countries, findings in mainland China may also be applicable across Asia and perhaps also for Asian immigrants in Western countries. The present study also has a number of limitations. First, the oversampling for women (60.9% of total study sample) may lead to a sex and health status selection bias. Particularly, healthy middle-aged men may have been less likely to respond to the survey. Unfortunately, no data were available for those who refused to participate in the study. To look for a potential sex bias among those who participated in the study, we analyzed the rates of undiagnosed diabetes and hypertension. A comparison analysis showed no sex-related difference in the rate of undiagnosed diabetes in middle-aged participants, but a higher rate of undiagnosed hypertension appeared in men of both age groups in the present study sample. Second, the data for the history of CVDs, family history of disease, income, exercise, and diet status were self-reported. A recalling bias may still appear though the self-reported CVDs were collected by a strict definition. Third, cases of CVD were identified only according to hospitalization for myocardial infarction, balloon angioplasty, cardiac stenting, and coronary artery bypass surgery, but not for angina and arrhythmias. Thus, the prevalence of CVD may be underestimated. Lastly, in this cross-sectional study, we could not investigate the causal relationship between lifestyle factors, risk factors for CVDs, and the development of diabetes in middle-aged men. Prospective data for this population will enable us to examine the interactions between lifestyle, CVD risk factors, and the incidences of diabetes and to test whether baseline disparities in healthy behavior and CVD risk factors between men and women predict future differences in CVD-related mortality.

In conclusion, our study provides evidence that the prevalence rates of diabetes, CVDs, and CVD risk factors are higher in men than women among the middle-aged Chinese population. In order to control and reduce modifiable risk factors for diabetes and CVDs, special prevention strategies and implementation plans tailored for men are urgently needed.

## Supporting Information

S1 Fig Prevalence of diabetes, CVD and CVD risk factors in the studied population. Prevalence of diabetes, CVD, CVD risk factors and their 95% confidence intervals (bar) in a Chinese population-based sample aged 30–40 years 3971 men (dot) and 6286 women (grey), and aged 41–50 years 4349 men and 7076 women. ‡ P < 0.001 for comparison between men and women.
(TIF)

S2 Fig Prevalence of family history of diabetes and related risk factors in the studied population. Prevalence of family history of diabetes, myocardial infarction, stroke, CVD, hypertension and their 95% confidence intervals (bar) in a Chinese population-based sample aged 30–40 years 3971 men (dot) and 6286 women (grey) and aged 41–50 years 4349 men and 7076 women. All P >0.05 for comparison between men and women.
(TIF)

## Abbreviations:

CVD: cardiovascular disease
CHD: coronary heart disease
CT: computed tomography
MRI: magnetic resonance imaging
CI: confidence interval
