# Supplementary figures and images for: Sex-Specific Prevalence of Diabetes and Cardiovascular Risk Factors in the Middle-Aged Population of China: A Subgroup Analysis of the 2007–2008 China National Diabetes and Metabolic Disorders Study
Source: PLoS One. 2015 Sep 25;10(9):e0139039. doi: 10.1371/journal.pone.0139039 (PMC4583471; doi:10.1371/journal.pone.0139039)

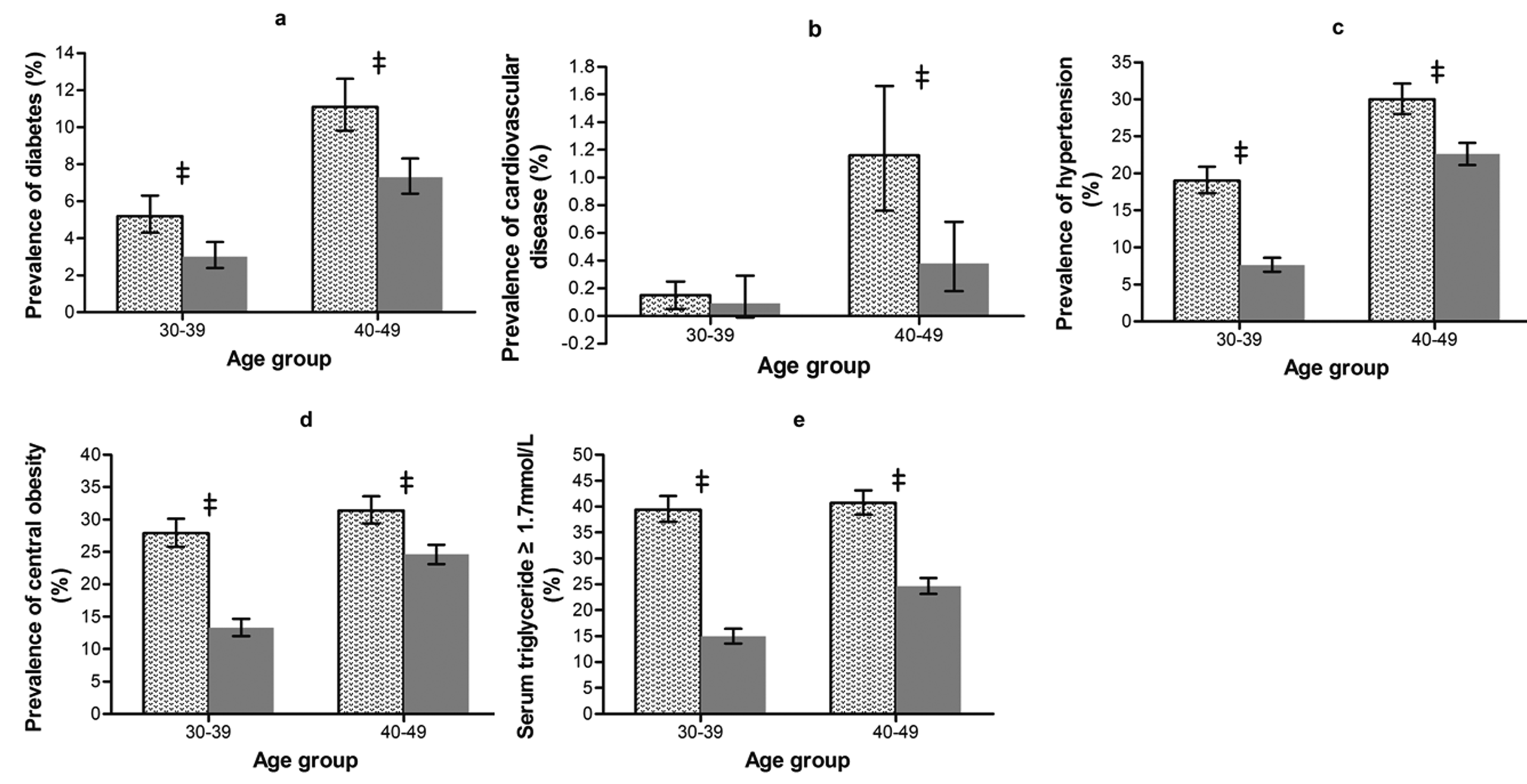

Supplement: S1 Fig — Prevalence of diabetes, CVD, CVD risk factors and their 95% confidence intervals (bar) in a Chinese population-based sample aged 30–40 years 3971 men (dot) and 6286 women (grey), and aged 41–50 years 4349 men and 7076 women. ‡ P < 0.001 for comparison between men and women. (TIF) [file pone.0139039.s001.tif]

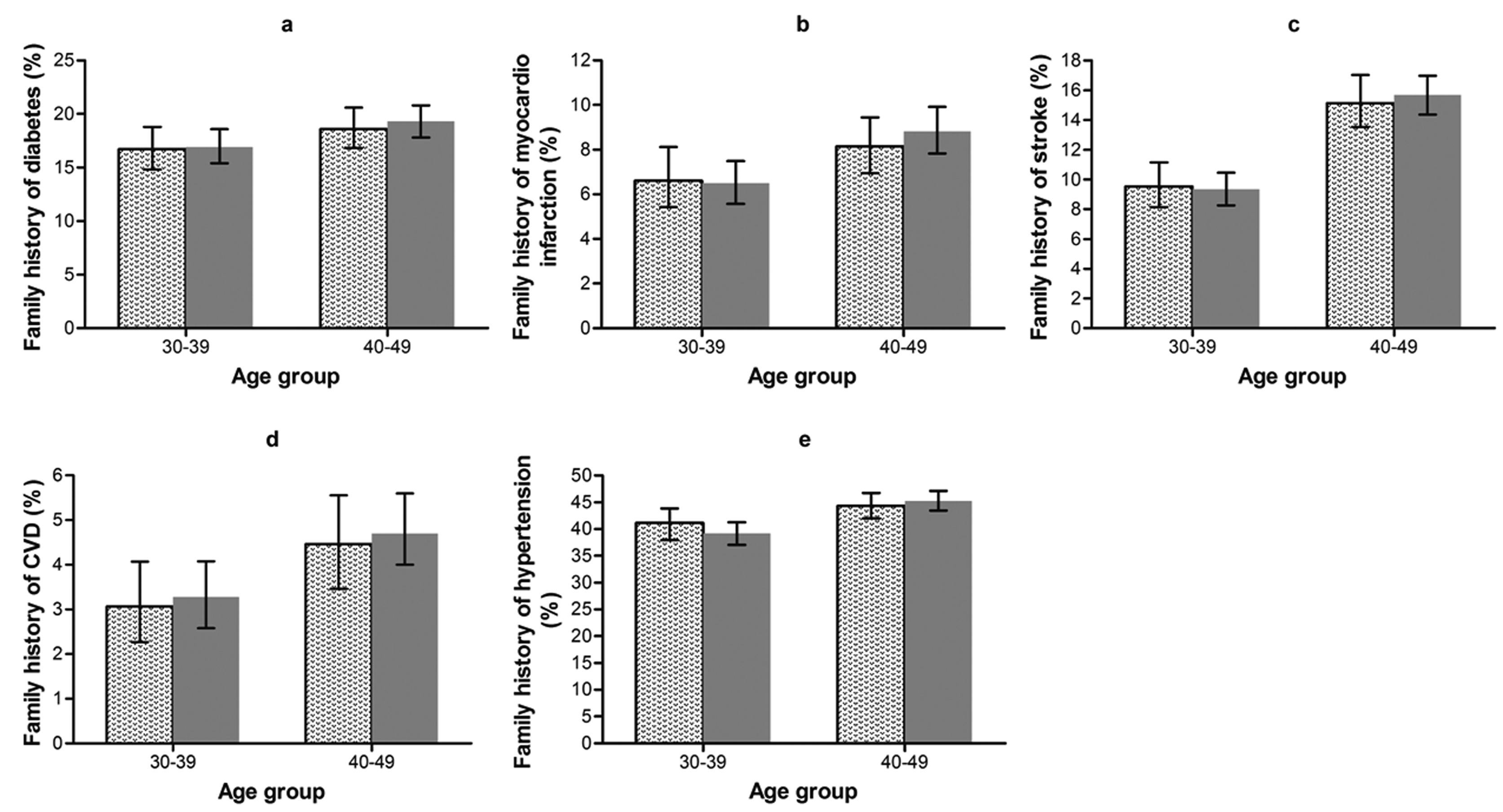

Supplement: S2 Fig — Prevalence of family history of diabetes, myocardial infarction, stroke, CVD, hypertension and their 95% confidence intervals (bar) in a Chinese population-based sample aged 30–40 years 3971 men (dot) and 6286 women (grey) and aged 41–50 years 4349 men and 7076 women. All P >0.05 for comparison between men and women. (TIF) [file pone.0139039.s002.tif]
